# Salvage endoscopic intermuscular dissection: curative treatment for residual rectal adenocarcinoma after chemoradiotherapy

**DOI:** 10.1055/a-2598-5198

**Published:** 2025-06-18

**Authors:** Francisca Côrte-Real, Nuno Nunes, Ana Rita Silva, Nadine Amaral, Ana Catarina Rego, Maria Antónia Duarte

**Affiliations:** 1 Hospital do Divino Espírito Santo de Ponta Delgada, Ponta Delgada, Portugal

We report the case of a 53-year-old male patient who underwent a total colonoscopy due to hematochezia and tenesmus. A circumferential vegetative lesion measuring approximately 50 mm in diameter, located 6 cm from the anal margin, was observed. Histology confirmed a moderately differentiated adenocarcinoma. The patient had a history of acute myocardial infarction 6 months earlier. Laboratory tests showed no anemia, and carcinoembryonic antigen (CEA) levels were within the normal range.

Pelvic magnetic resonance imaging (MRI) staged as T3bN0, with extramural vascular invasion (EMVI), without involvement of the mesorectal fascia. Computed tomography ruled out metastases.

The patient underwent neoadjuvant chemoradiotherapy, achieving a complete response on follow-up pelvic MRI. However, rectosigmoidoscopy revealed a flat lesion with central ulceration, measuring 20 mm ( Fig. 1 ). Histology confirmed the presence of adenocarcinoma.

**Fig. 1:**
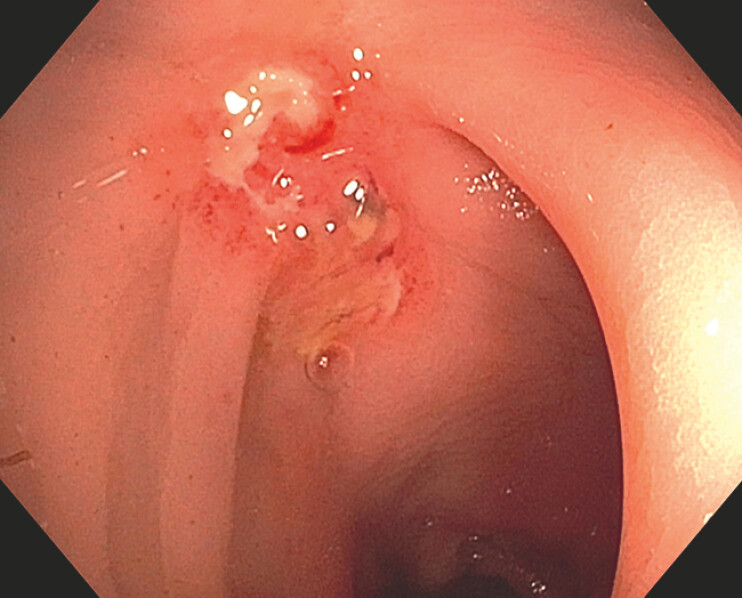
Residual rectal adenocarcinoma.

The patient refused surgery. Following a discussion in a multidisciplinary team meeting, he underwent endoscopic intermuscular dissection of the lesion ( Video 1 ). Histology demonstrated an R0 resection of a moderately differentiated adenocarcinoma invading the submucosa, with preservation of the muscularis propria. No lymphovascular invasion, perineural invasion, or tumor budding was observed.

Endoscopic intermuscular dissection of the residual rectal lesion after chemoradiotherapy.Video 1

Endoscopic intermuscular dissection is widely used for the management of rectal lesions suspected of deep submucosal invasion. This technique allows for complete lesion excision and represents an emerging strategy in the treatment of rectal adenocarcinoma 1 .

This case underscores the relevance of endoscopic dissection in the management of colorectal lesions, including those under the watchful waiting approach. It could be used as a salvage method, potentially avoiding abdominoperineal amputation. The patient remains asymptomatic and under surveillance, with no evidence of recurrence at 20-month follow-up ( Fig. 2 ) 2 3 .

**Fig. 2:**
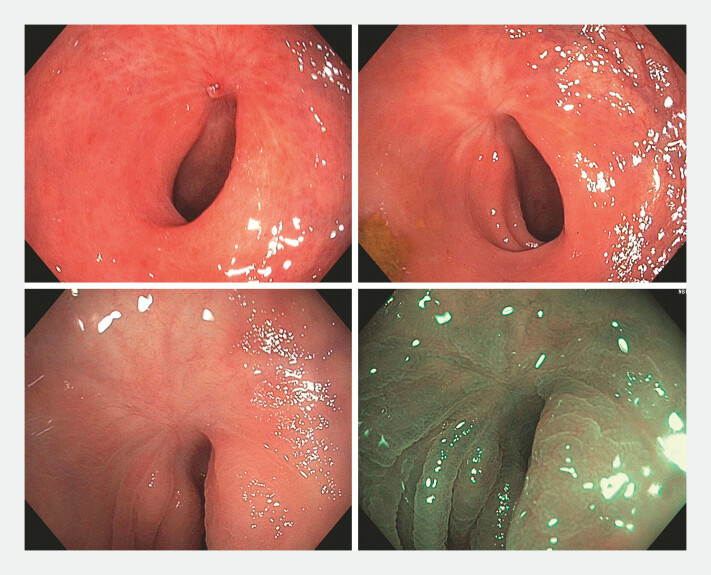
Surveillance rectosigmoidoscopies without recurrence.

Endoscopy_UCTN_Code_TTT_1AQ_2AC

## Notes

Endoscopy E-Videos https://eref.thieme.de/e-videos
*E-Videos* is an open access online section of the journal *Endoscopy* , reporting on interesting cases and new techniques in gastroenterological endoscopy. All papers include a high-quality video and are published with a Creative Commons CC-BY license. Endoscopy E-Videos qualify for HINARI discounts and waivers and eligibility is automatically checked during the submission process. We grant 100% waivers to articles whose corresponding authors are based in Group A countries and 50% waivers to those who are based in Group B countries as classified by Research4Life (see: https://www.research4life.org/access/eligibility/ ). This section has its own submission website at https://mc.manuscriptcentral.com/e-videos .
